# Suppression of Type I Interferon Signaling in Myeloid Cells by Autoantibodies in Severe COVID-19 Patients

**DOI:** 10.1007/s10875-024-01708-7

**Published:** 2024-04-22

**Authors:** Ami Aoki, Chiaki Iwamura, Masahiro Kiuchi, Kaori Tsuji, Atsushi Sasaki, Takahisa Hishiya, Rui Hirasawa, Kota Kokubo, Sachiko Kuriyama, Atsushi Onodera, Tadanaga Shimada, Tetsutaro Nagaoka, Satoru Ishikawa, Akira Kojima, Haruki Mito, Ryota Hase, Yasunori Kasahara, Naohide Kuriyama, Sukeyuki Nakamura, Takashi Urushibara, Satoru Kaneda, Seiichiro Sakao, Osamu Nishida, Kazuhisa Takahashi, Motoko Y. Kimura, Shinichiro Motohashi, Hidetoshi Igari, Yuzuru Ikehara, Hiroshi Nakajima, Takuji Suzuki, Hideki Hanaoka, Taka-aki Nakada, Toshiaki Kikuchi, Toshinori Nakayama, Koutaro Yokote, Kiyoshi Hirahara

**Affiliations:** 1 Department of Immunology, Graduate School of Medicine, Chiba University, Chiba, 260-8670 Japan; 2 Department of Respiratory Medicine and Infectious Diseases, Niigata University Graduate School of Medical and Dental Sciences, Niigata, 951-8510 Japan; 3 Synergy Institute for Futuristic Mucosal Vaccine Research and Development, Chiba University, Chiba, Japan; 4 Department of Emergency and Critical Care Medicine, Graduate School of Medicine, Chiba University, Chiba, 260-8670 Japan; 5 Department of Respiratory Medicine, Juntendo University Faculty of Medicine and Graduate School of Medicine, Tokyo, 113-8431 Japan; 6 Funabashi Central Hospital, Chiba, 273-8556 Japan; 7 Department of Infectious Diseases, Japanese Red Cross Narita Hospital, Chiba, 286-0041 Japan; 8 Department of Respiratory Medicine, Eastern Chiba Medical Center, Chiba, 283-8686 Japan; 9 Department of Anesthesiology and Critical Care Medicine, School of Medicine, Fujita Health University, Toyoake, Aichi 470-1192 Japan; 10 Funabashi Municipal Medical Center, Chiba, 273-8588 Japan; 11 Kimitsu Chuo Hospital, Chiba, 292-0822 Japan; 12 Department of Gastroenterology, NHO Chiba Medical Center, Chiba, 260-8606 Japan; 13 Department of Pulmonary Medicine, International University of Health and Welfare Narita Hospital, Chiba, 286-8520 Japan; 14 Department of Experimental Immunology, Graduate School of Medicine, Chiba University, Chiba, 260-8670 Japan; 15 Department of Medical Immunology, Graduate School of Medicine, Chiba University, Chiba, 260-8670 Japan; 16 Department of Infectious Diseases, Chiba University Hospital, Chiba, 260-8677 Japan; 17 COVID-19 Vaccine Center, Chiba University Hospital, Chiba, 260-8677 Japan; 18 Department of Pathology, Graduate School of Medicine, Chiba University, Chiba, 260-8670 Japan; 19 Department of Allergy and Clinical Immunology, Graduate School of Medicine, Chiba University, Chiba, 260-8670 Japan; 20 Department of Respirology, Graduate School of Medicine, Chiba University, Chiba, 260-8670 Japan; 21 Clinical Research Center, Chiba University Hospital, Chiba, 260-8677 Japan; 22 AMED-CREST, AMED, 1-8-1 Inohana, Chuo-ku, Chiba, 260-8670 Japan; 23 Department of Endocrinology, Hematology and Gerontology, Graduate School of Medicine, Chiba University, Chiba, 260-8670 Japan

**Keywords:** COVID-19, Type I IFNs, Autoantibody, Single-cell RNA sequencing, BCR repertoires, Epitope mapping

## Abstract

**Purpose:**

Auto-antibodies (auto-abs) to type I interferons (IFNs) have been identified in patients with life-threatening coronavirus disease 2019 (COVID-19), suggesting that the presence of auto-abs may be a risk factor for disease severity. We therefore investigated the mechanism underlying COVID-19 exacerbation induced by auto-abs to type I IFNs.

**Methods:**

We evaluated plasma from 123 patients with COVID-19 to measure auto-abs to type I IFNs. We performed single-cell RNA sequencing (scRNA-seq) of peripheral blood mononuclear cells from the patients with auto-abs and conducted epitope mapping of the auto-abs.

**Results:**

Three of 19 severe and 4 of 42 critical COVID-19 patients had neutralizing auto-abs to type I IFNs. Patients with auto-abs to type I IFNs showed no characteristic clinical features. scRNA-seq from 38 patients with COVID-19 revealed that IFN signaling in conventional dendritic cells and canonical monocytes was attenuated, and SARS-CoV-2-specific BCR repertoires were decreased in patients with auto-abs. Furthermore, auto-abs to IFN-α2 from COVID-19 patients with auto-abs recognized characteristic epitopes of IFN-α2, which binds to the receptor.

**Conclusion:**

Auto-abs to type I IFN found in COVID-19 patients inhibited IFN signaling in dendritic cells and monocytes by blocking the binding of type I IFN to its receptor. The failure to properly induce production of an antibody to SARS-CoV-2 may be a causative factor of COVID-19 severity.

**Supplementary Information:**

The online version contains supplementary material available at 10.1007/s10875-024-01708-7.

## Introduction

Coronavirus disease 2019 (COVID-19) is an infectious disease caused by severe acute respiratory syndrome coronavirus 2 (SARS-CoV-2). Although most COVID-19 patients show mild disease, some suffer from severe symptoms, leading to death due to severe respiratory failure and thromboembolism [1]. Prediction of the severity of COVID-19 in advance is thus critical to provide appropriate medical resources to severe patients and hopefully improve their prognosis.

Factors that contribute to the severity of COVID-19 include the patient’s age and gender as well as existence of underlying medical conditions [2–7]. However, COVID-19 can be severe even in the absence of well-known factors, suggesting that there are other factors that cause COVID-19 to be severe. Patients with COVID-19 have been reported to have autoantibodies against various extracellular or secreted proteins [8]. Particularly autoantibodies against cytokines are implicated in the pathogenesis and severity of infectious diseases including COVID-19 [8, 9].

An innate immune response by type I interferons (IFNs) is essential for the host defense against SARS-CoV-2 in the early phase of infection [10–12]. Recent reports have also revealed that genetic defects in IFN signaling contribute to life-threatening COVID-19 [13–21]. Therefore, a lack of IFN signaling can be a factor inducing severe COVID-19.

Prior to the COVID-19 pandemic, auto-antibodies (abs) to type I IFNs were detected only in a limited number of patients with severe chickenpox infection and systematic lupus erythematosus (SLE), or auto-immune polyendocrinopathy syndrome type 1 (APS-1) [22, 23]. However, about 10.2% of life-threatening COVID-19 cases have been found to have auto-abs to type I IFNs, indicating the involvement of auto-abs to type I IFNs in the severity of COVID-19 [13, 24–31].

To clarify the effects of auto-abs to type I IFNs on the pathogenesis of severe COVID-19, we investigated the immune profiles of patients with auto-abs to type I IFNs and the detailed characteristics of the auto-abs (Fig. S1).

## Methods

### Subjects and Samples

A total of 123 patients confirmed by RT-PCR to have been infected with SARS-CoV-2 from late July 2020 to March 2021 were included in the study. None of them were immunized with COVID-19 vaccines. The inclusion criteria were patient age ≥20 years old, with other no specific exclusion criteria aside from age. Patient severity was classified according to a previous report [32]: Mild COVID-19 patients are individuals with SARS-CoV-2 infection confirmed by a PCR test, who did not require oxygen supplementation with or without evidence of pneumonia during hospitalization. Severe COVID-19 was defined as patients requiring low-flow oxygen (<6 liters / min). Critical COVID-19 was defined as patients requiring high-flow oxygen, mechanical ventilation. septic shock, or with damage to any other organ requiring admission to the intensive care unit.

We retrospectively evaluated and analyzed the detailed medical history, physical examination findings, and hematological and biochemical evaluation results obtained from the patients. Blood samples were collected using EDTA-2Na blood collection tubes once every week for mild and severe cases or two to three times per week for critical cases. Peripheral blood mononuclear cells (PBMCs) were separated using Ficoll-Paque® density gradient centrifugation. The samples were aliquoted and then stored at -80 °C. The details of the enrolled patients are described in our previous study [33].

### An ELISA for Measuring Auto-Abs to Type I IFNs in Plasma

The concentrations of auto-abs to type I IFNs in the plasma of COVID-19 patients were measured using ELISAs as previously described [34]. In brief, on the day before the experiment, 96-well plates were coated with 0.5 μg/ml of *E. coli* derived non-glycosylated recombinant human (rh) IFN-α2 (11101-1; R&D systems). After washing with PBS (0.05% Tween) and blocking (UKB80; KAC Co., Ltd.), patient plasma diluted 1:50 or rabbit anti-human IFN-α2 IgG as a positive control (ab193055; Abcam) was added to the plate. After washing, auto-abs captured by rh type I IFNs were detected by peroxidase-labeled anti-human IgG antibody (62-8420; Invitrogen) or anti-rabbit IgG (H+L) antibody (G-212340; Invitrogen). Color was developed by TMB substrate (1721066; Bio-Rad), and absorbance values were measured at 450 nm with a plate reader (Bio-Rad).

### The Evaluation of the Neutralizing Capacity of Auto-Abs to Type I IFNs

The neutralizing ability of auto-abs to type I IFNs was determined by evaluating the inhibition of STAT1 phosphorylation in human monocyte-derived U937 cell lines (Japan Collection Research Bioresources Cell Bank) as previously described [34]. The U937 cells were adjusted to 5 × 10^4^ cells/40 μl and mixed with 5 μl of plasma (1:10 dilution), rhIFN-α2 (11101-1; R&D), rhIFN-β (8499-IF; R&D), or rhIFN-ω (11395-1; R&D) (final concentration 10 ng/ml). These supplemented IFNs were *E. coli* derived non-glycosylated proteins. After incubation at 37 °C for 15 min, the cells were fixed with 4% PFA for 10 min at room temperature and permeabilized with 90% MetOH at -20 °C for 1h. After washing, the cells were stained with Alexa Fluor 647-labeled anti-pSTAT1 (pY701) antibody (562070; BD Biosciences) and incubated overnight at 4 °C. The phosphorylation of pSTAT1 in U937 cells was analyzed by a flow cytometer (BD Biosciences).

### Single Cell RNA-Sequencing (scRNA-seq) for Gene Expressions and B Cell Receptor (BCR) Repertoire Analyses

PBMC samples recovered from mild, severe and critical COVID-19 patients were stained with anti-human CD45 antibody. Live CD45^+^ cells and CD45^+^CD19^+^ cells (3,000-10,000 cells each, cell viability >98%) were sorted using a cell sorter (SH800S; SONY) and then encapsulated into droplets for gene expression and BCR repertoire analyses. Libraries for the analyses were prepared using Chromium Single Cell 5’ Reagent Kits v1.1 and Chromium Single Cell Human BCR Amplification Kit by following the manufacturer’s protocol (10X Genomics). The generated scRNA-seq and sc V(D)J -seq libraries were sequenced using a total of 308 cycles (paired-end reads) with a NovaSeq 6000 sequencer (Illumina, Inc.).

### scRNA-seq Analyses

Sequence reads from all samples were processed and aggregated into FASTQ file using a Cell Ranger v6.0.0 (10x genomics). Aggregated data were further analyzed by Seurat v4.1.0 [35]. Specifically, we log-normalized the expression matrix, regressed the data against the total number of unique molecular identifiers (UMIs) detected per cell, performed a principal component analysis (PCA), used PCA dimensions 1-50 to find clusters on a uniform manifold approximation and projection (UMAP), and visualized the single-cell gene expression as UMAP overlays, violin plots, heat maps, and dot plots by Seurat. Single-cell gene expressions were also visualized as density plots using the Nebulosa R software package (version 1.0.1) algorithm [36]. We detected the activated pathways by a single-sample gene set variation analysis (ssGSVA v1.49.1). The ssGSVA scores were visualized with UMAP overlays, ridgeline plots, and ranking plots. scV(D)J -seq assembled BCR V(D)J sequences from FASTQ files using the Cellranger vdj pipeline and analyzed paired chronotype calling. Then, Chromium cellular barcodes and UMI were used to assemble cell-specific V(D)J transcripts and identify BCR clonotypes and CDR3 sequences. BCR clones were counted for amino acid sequence homology duplications in CDR3 and assigned by R to five clone size categories: single (0 < x < = 1), small (1 < x < = 5), media, (5 < x < = 20) large (20 < x < = 50) and extra-large (x > 50). Bar graph of clone size was drawn by ggplot2 v3.4.2 (https://ggplot2.tidyverse.org/). To define ISGs, we referred to an article published by van der Wijst et al [37]. Diversity index was calculated by following the studies reported by Ye et al [38] and Wang et al [39].

### Epitope Mapping

A total of 146 amino acid sequences of 20 peptide chains of human IFN-α2 (NP 000596.2, 188aa) overlapping by 1 amino acid residue were designed. These peptides were synthesized by JPT Peptide Technologies. The synthetic peptides were immobilized on glass slides by the Pepstar method and reacted with patient plasma. Diluted plasma (1/200) from patients with auto-abs to IFN-α2 (*n*=7), plasma from patients without auto-abs to IFN-α2 (*n*=6) and healthy subjects (*n*=10) were tested. After binding with IgG secondary antibody, the signal intensity was detected to evaluate the affinity intensity of IgG from patient plasma to the IFN-α2 epitope. Finally, 146 amino acid sequences were analyzed by linking them to the three-dimensional (3D) structure of IFN-α2 using Waals software (Altif Laboratories Inc.).

### ELISA for IFN-α2 Peptides

IFN-α2_80-95_ and IFN-α2_124-143_ peptides were synthetized by Cosmo Bio Co., Ltd. For effective binding to a plate, cysteine was added to the N-terminal of IFN-α2_80-95_, and amino residue was conjugated to the C-terminal. Biotin was conjugated to the N-terminal of IFN-α2_124-143_. Immobilizer amino (436006; Thermo Fisher Scientific Inc.) or Immobilizer streptavidin (436014; Thermo Fisher Scientific Inc.) 96 well plate was coated with 10 μg/ml of each peptide. After washing with PBS (0.05% Tween) and blocking with 10 mM Ethanolamine (15014; Sigma Aldrich) for Immobilizer amine plates, undiluted patient plasma was added to the wells. After washing, auto-abs captured by the peptides were detected by peroxidase-labeled anti-human IgG antibody (62-8420; Invitrogen). Color was developed by TMB substrate (1721066; Bio-Rad), and absorbance values were measured at 450 nm with a plate reader (Bio-Rad).

### Measurement of SARS-CoV-2-Specific Antibody

Antibody titters to S protein of SARS-CoV-2 in the patient’s plasma were measured by SRL, Inc. using Elecsys anti-SARS-CoV-2 S RUO (Roche Diagnostics K.K.). Values >0.8 U/mL were determined to be positive.

### Statistical Analyses

The comparison of proportions was performed using a chi-square test. Statistical significance was assessed using an unpaired two-tailed Mann-Whitney U-test for two groups and Wilcoxon’s matched-pairs signed rank test. One-way ANOVA was used for multiple comparison. All statistical analyses were performed using the Prism GraphPad Software program.

## Results

### Detection of Neutralizing Auto-Abs to Type I IFNs in Patients with Severe and Critical COVID-19

**Fig. 1:**
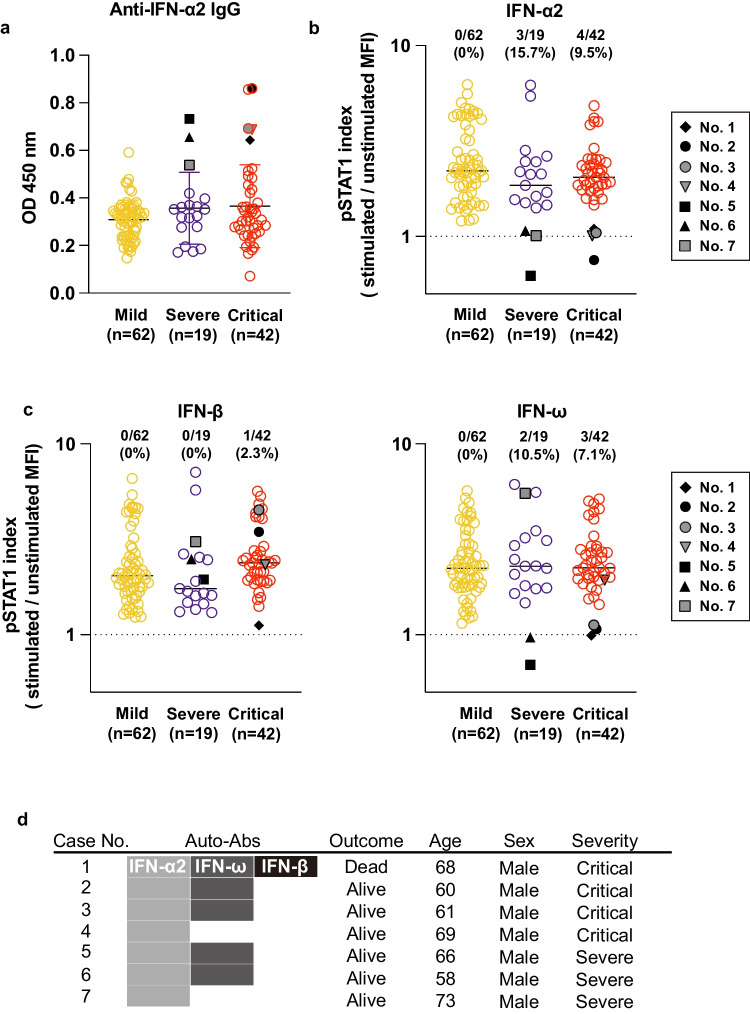
Detection of neutralizing auto-abs to IFN-α2, IFN-β, and IFN-ω in severe or critical COVID-19 patients. **a**. Measurement of auto-abs to human IFN-α2 in the plasma from mild, severe and critical COVID-19 patients using an ELISA. Black or gray circles, diamond, triangles, and rectangles represent patients carrying anti-IFN-α2 antibodies (*n*=7). **b**. Neutralizing activity of the auto-abs to IFN-α2. The phosphorylation of STAT1 in U937 cells stimulated with recombinant human IFN-α2 was evaluated in the presence of 10% plasma from patients (*n*=123). Black or gray circles, diamond, triangles, and rectangles represent patients carrying neutralizing anti-IFN-α2 antibodies (*n*=7). **c.** Neutralizing activity of auto-abs to IFN-β or IFN-ω. Phosphorylation of STAT1 in U937 cells stimulated with recombinant human IFN-β or IFN-ω was evaluated in the presence of 10% plasma from patients (*n*=123). **d**. Characteristics of each patient with neutralizing auto-abs to type I IFNs

We first performed an ELISA to determine if patients with COVID-19 had auto-abs to type I IFNs in their plasma. We found that 3 of 19 severe, 7 of 42 critical and 1 of 62 mild COVID-19 patients had elevated titers of IgG to IFN-α2 (defined as levels ≧ 0.5 OD 450nm) (Fig. 1a). To investigate whether or not the auto-abs in the patients with COVID-19 had a neutralizing ability against type I IFNs, we stimulated the U937 cell line with recombinant IFN-α2 in the presence of plasma from patients with COVID-19. Supplementation of plasma from the seven patients with auto-abs to type I IFN-α2 resulted in the decreased phosphorylation of signal transducers and activations of transcription 1 (STAT1) in U937 cells, which indicated that the auto-abs in the patients had neutralizing activity to IFN-α2 (Fig. 1b). Plasma from five out of the seven patients who had auto-abs to IFN-α2 also inhibited the IFN-ω-induced phosphorylation of STAT1 in U937 cells (Fig. 1c). Furthermore, the plasma of one patient who had auto-abs to IFN-α2 inhibited both the IFN-ω- and IFN-β-induced phosphorylation of STAT1 (Fig. 1c). When phosphorylation of STAT1 was assessed using plasma from healthy subjects and COVID-19 patients, the levels were comparable between the two groups (Fig. S2). The patients who had auto-abs to IFN-α2, IFN-β, and IFN-ω died with COVID-19, whereas the other patients who had auto-Ab to IFN-α2 alone or auto-abs to IFN-α2 and IFN-β survived (Fig. 1d). Of the 123 cases analyzed in this study, 12 patients were deceased, one of which harbored auto-abs to IFNs. All patients with auto-abs to type I IFNs were male with a mean age of 65 years old (Fig. 1d and Table 1).

**Table 1:** Clinical characteristics of severe and critical COVID-19 patients with and without auto-abs

			Anti-type I IFN abs (-)	Anti-type I IFN abs (+)	*P* value
Number			54	7	
Age					
	Mean (SD)		60.0 (12.7)	65.0 (5.5)	0.31
	Distribution,	<65 years	61.1 (33/54)	42.9 (3/7)	0.36
	% (n/total n)	≥65 years	38.9 (21/54)	57.1 (4/7)	
Sex			% (n/total n)	% (n/total n)	
	Male		83.3 (45/54)	100 (7/7)	0.24
	Female		16.7 (9/54)	0 (0/7)	0.24
BMI					
	Median (IQR)		26.5 (24.4-29.8)	25.2 (24.0-26.3)	0.29
	Distribution, % (n/total n)	<30.0	75.8 (25/33)	100 (6/6)	0.18
	% (n/total n)	≥30.0	24.2 (8/33)	0 (0/6)	0.18
Previous history			% (n/total n)	% (n/total n)	
	Hepatitis		3.7 (2/54)	0 (0/7)	0.60
	Cancer		3.7 (2/54)	0 (0/7)	0.60
	Heart disease		9.3 (5/54)	14.3 (1/7)	0.53
	Cerebral infarction		9.3 (5/54)	0 (0/7)	0.4
	Infection				
		Syphilis	1.9 (1/54)	0.0 (0/7)	0.71
		HTLV-1	1.9 (1/54)	0.0 (0/7)	0.71
		Severe infection	0 (0/54)	0 (0/7)	
Comorbidities			% (n/total n)	% (n/total n)	
	None		14.8 (8/54)	14.3 (1/7)	0.97
	Hypertension		42.6 (23/54)	14.3 (1/7)	0.15
	Diabetes	All	39.0 (21/54)	42.9 (3/7)	0.72
		Type 2	37.0 (20/54)	42.9 (3/7)	0.77
		Type 1	1.9 (1/54)	0.0 (0/7)	0.71
	Dyslipidemia		29.6 (16/54)	42.9 (3/7)	0.40
	Pulmonary diseases		18.5 (10/54)	14.3 (1/7)	0.78
		Asthma	7.4 (4/54)	14.3 (1/7)	0.53
		Interstitial lung disease	3.7 (2/54)	14.3 (1/7)	0.60
		Chronic obstructive pulmonary disease	7.4 (4/54)	0 (0/7)	0.60
		Sleep apnea syndrome	5.6 (3/54)	0 (0/7)	0.52
	Chronic renal failure		9.3 (5/54)	0 (0/7)	0.4
		Maintenance dialysis	7.4 (4/54)	0 (0/7)	0.46
	Cardiovascular disease		11.1 (6/54)	0 (0/7)	0.35
		Arrhythmia	7.4 (4/54)	0 (0/7)	0.60
		Vascular disease	1.9 (1/54)	0 (0/7)	0.71
		Heart failure	1.9 (1/54)	0 (0/7)	0.71
		Dissecting aortic aneurysm	1.9 (1/54)	0 (0/7)	0.71
	Endocrine disease		5.6 (3/54)	0 (0/7)	0.52
		Hypothyroidism	1.9 (1/54)	0 (0/7)	0.71
		Primary aldosteronism	1.9 (1/54)	0 (0/7)	0.71
		Panhypopituitarism	1.9 (1/54)	0 (0/7)	0.71
	Myasthenia gravis		0 (0/54)	14.3 (1/7)	0.0051
	Dementia		3.7 (2/54)	0 (0/7)	0.60
Mortality			% (n/total n)	% (n/total n)	
	Deceased		20.4 (11/54)	14.3 (1/7)	0.7

The chi-square test was used to analyze the effect of dichotomous variables, while the Mann-Whitney U test was used for continuous variables

We ultimately found that 7 out of 123 Japanese patients with COVID-19 (5.7%) had neutralizing auto-abs to IFN-α2. Of note, all seven cases suffered from severe or critical COVID-19 and the proportions of patients with auto-abs to IFN-α2 among the severe or critical COVID-19 patients were 15.7% or 9.5% respectively.

### Absence of Unique Clinical Features in Patients with Auto-Abs to Type I IFN

To determine the clinical characteristics of patients with and without type I IFN auto-abs, we examined the clinical data of each patient (Table 1). None of the patients with auto-abs had a history of severe infections (e.g. influenza or varicella zoster virus) or connective tissue diseases (e.g. systemic lupus erythematosus or rheumatoid arthritis), had been treated with IFNs for chronic infection of hepatitis C or had adverse reaction to vaccine against yellow fever virus (YFV-17D) [21, 32, 40, 41]. One of the patients with auto-abs to IFN-α2, IFN-β, and IFN-ω (Case no. 1) suffered from myasthenia gravis. The medical history as well as the body mass index (BMI) were similar between the patients with and without auto-abs to type I IFNs (Table 1).

Regarding the treatment against COVID-19, antiviral, anti-inflammatory, or anticoagulant drugs were administered to severe or critical patients, regardless of the presence of anti-IFN auto-abs (Table 2). The frequency of ventilator or extracorporeal membrane oxygen (ECMO) therapies, the incidence of complications, and the mortality rate were similar between the two groups (Table 2). The blood level of lactate dehydrogenase (LDH) in the patients with anti-IFN auto-abs was significantly lower than in the patients without anti-IFNs auto-abs. The number of white blood cells, neutrophils, and monocytes tended to be higher in the patients with auto-abs to type I IFNs than in those without auto-abs, but these differences were not statistically significant (Table 3).

**Table 2:** The clinical course of severe and critical COVID-19 patients with and without auto-abs

			Anti-type I IFN abs (-)	Anti-type I IFN abs (+)	*P* value
Treatment			% (n/total n)	% (n/total n)	
	Anti-viral		87.5 (49/54)	100 (7/7)	0.4
		Remdesivir	66.7 (36/54)	71.4 (5/7)	0.8
		Favipiravir	53.7 (29/54)	71.4 (5/7)	0.37
	Anti-coagulants		70.4 (38/54)	71.4 (5/7)	0.95
	Anti-biotics		50.0 (27/54)	28.6 (2/7)	0.29
	Anti-fungi		13.0 (7/54)	0 (0/7)	0.31
	Glucocorticoids		94.4 (51/54)	100 (7/7)	0.52
	Tocilizumab		27.8 (15/54)	28.6 (2/7)	0.96
Critical care			% (n/total n)	% (n/total n)	
	Oxygen		98.2 (53/54)	100 (7/7)	0.72
	Mechanical ventilation		57.4 (31/54)	57.1 (4/7)	0.99
	ECMO		20.4 (11/54)	14.3 (1/7)	0.70
	Hemoperfusion		1.9 (1/54)	0 (0/7)	0.72
	Continuous Hemodiafiltration		22.2 (12/54)	14.3 (1/7)	0.63
	Plasma exchange		9.3 (5/54)	14.3 (1/7)	0.67
Complications			% (n/total n)	% (n/total n)	
	None		75.9 (41/54)	57.1 (4/7)	0.29
	Complications		24.1 (13/54)	42.9 (3/7)	0.29
	Co-infection		13.0 (7/54)	28.6 (2/7)	0.27
	Hemorrhaging		7.4 (4/54)	0 (0/7)	0.46
	Guillain-Barre syndrome		1.9 (1/54)	0 (0/7)	0.72
	Pneumothorax/Mediastinal emphysema		5.6 (3/54)	14.3 (1/7)	0.38
	Thrombocytopenia		9.3 (5/54)	0 (0/7)	0.4
	Disseminated intravascular coagulation		5.6 (3/54)	0 (0/7)	0.52
	Nonocclusive mesenteric ischemia		1.9 (1/54)	0 (0/7)	0.72
	Pancreatitis		1.9 (1/54)	0 (0/7)	0.72
	Acute renal infarction	KDIGO: eGFR < 60 /min	38.9 (21/54)	42.9 (3/7)	0.84
	Diarrhea		0 (0/54)	14.3 (1/7)	0.0051
	Takotsubo cardiomyopathy		0 (0/54)	14.3 (1/7)	0.0051
	Skin rash		0 (0/54)	14.3 (1/7)	0.0051

*P* values were calculated using the chi-squared test

*KDIGO* Kidney Disease Improving Global Outcomes, *eGFR* estimated Glomerular Filtration Rate

**Table 3:** Laboratory data of severe and critical COVID-19 patients with and without auto-abs

Laboratory data	Anti-type I IFN abs (-)	Anti-type IFN abs (+)	*P* value
WBC, /μl, median (IQR)	7650 (5600-11525)	11000 (7500-13100)	0.10
Neutrophils, /μl, median (IQR)	6676 (4737-10020)	9625 (6034 -12013)	0.16
Lymphocytes, /μl, median (IQR)	678 (351-856)	511 (388-1091)	0.66
Monocytes, /μl, median (IQR)	312 (180-456)	563 (275-776)	0.13
Hemoglobin, g/dl, median (IQR)	13.3 (11.9-14.5)	13.7 (12.2-14.4)	0.62
Platelet, 1×10^3^/μl, median (IQR)	225 (156-266)	217 (178-276)	0.62
FDP, μg/ml, median (IQR)	6.8 (3.3-15.7)	3.9 (1.9-5.6)	0.20
CRP, mg/ml, median (IQR)	7.3 (5.3-13.4)	5.3 (3.8-20.4)	0.71
LDH, U/l, median (IQR)	407 (294-498)	264 (248-300)	0.014
Total Protein, g/dl, median (IQR)	6.0 (5.3-6.5)	5.7 (5.3-6.6)	0.83
Albumin, g/dl, median (IQR)	2.8 (2.5-3.2)	3.1 (2.5-3.4)	0.46
eGFR, ml/min/1.73 m^2^, median (IQR)	67.2 (41.2-87.8)	77.7 (64.1-96.9)	0.22

*P* values were calculated using Mann-Whitney U test

*WBC* White Blood Cell, *FDP* Fibrin/fibrinogen Degradation Products, *CRP* C-Reactive Protein, *LDH* Lactate Dehydrogenase, *eGFR* estimated Glomerular Filtration Rate

**Fig. 2:**
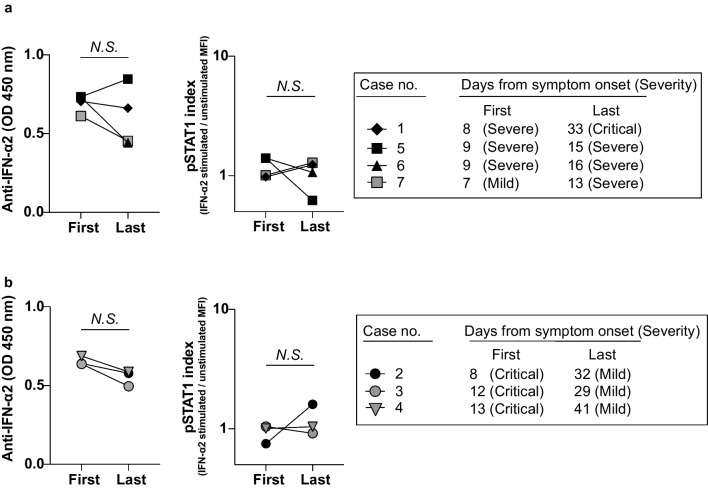
A longitudinal analysis of auto-abs titers and the neutralizing activity to IFN-α2 during hospitalization. **a**. Auto-abs to IFN-α2 titers and the neutralizing activity in four patients (Case No. 1, 5, 6, 7) who did not show improvement during hospitalization. **b**. Auto-abs to IFN-α2 titers and the neutralizing activity in three patients (Case No. 2, 3, 4) who recovered from COVID-19. *P* values were determined by Wilcoxon’s matched-pairs signed rank test

Regarding the auto-abs titer and neutralizing activity to IFN-α2 during hospitalization, the titers of auto-abs and neutralizing activity showed no significant difference between early after the onset and late during hospitalization among the patients who showed no clinical recovery (Fig. 2a). Interestingly, patients with clinical recovery also showed similar titers of auto-abs and neutralizing activity during hospitalization (Fig. 2b).

Thus, it is difficult to estimate the presence of anti-IFN abs from the usual blood tests and clinical background.

### Attenuation of IFN Signaling in Innate Cells by Auto-Abs to IFN-α2

**Fig. 3:**
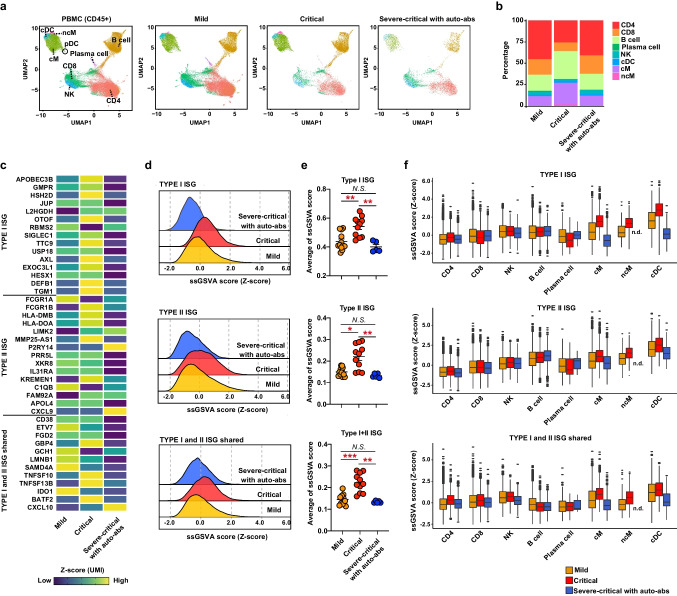
A scRNA-seq analysis for leukocytes composition and ISG signaling in COVID-19 patients with auto-abs to type IFNs. **a**. UMAP projections of CD45^+^ cells isolated from PBMCs of 28 COVID-19 patients (Mild: *n*=12, Critical: *n*=11, Severe or critical with auto-abs: *n*=5 [Case no. 1, 2, 3, 4 and 5]). Nine clusters were identified, including CD4^+^ T cells (CD4), CD8^+^ T cells (CD8), NK cells (NK), B cells, plasma cells, canonical monocytes (cM), non-canonical monocytes (ncM), conventional dendritic cells (cDC), and plasmacytoid DCs (pDC). **b**. Bar plots depicting immune cell-type composition in COVID-19 patients showing mild and severe or critical symptoms in addition to severe or critical patients carrying auto-abs to type I IFNs. **c.** Heatmap showing specific type I IFN-stimulated gene (ISG), type II ISG, and shared ISG-I and ISG-II in CD45^+^ cells from the three groups. **d.** Single-sample gene set variant analysis (ssGSVA) scores of types I and/or II ISG in each patient group. **e.** Average of ssGSVA scores of types I and/or II ISG in each patient. One-way ANOVA was used for the statistical analyses. Mean ± SEM. *p <0.05, **p <0.01, ***p <0.001. **f.** ssGSVA scores of types I and/or II ISG in each cell type of each group

To investigate the effect of neutralizing auto-abs to type I IFNs on immune responses in COVID-19 patients, we performed scRNA-Seq using PBMCs from COVID-19 patients. UMAP identified 9 clusters based on lineage-specific marker genes in PBMCs collected from 4 critical and 1 severe patients with neutralizing auto-abs to type I IFNs (Case no. 1, 2, 3, 4 and 5) and 11 critical patients without auto-abs to type I IFNs, as well as 12 patients with mild COVID-19 (Fig. 3a, Fig. S3a-b). The ratios of CD4^+^ T cells, CD8^+^ T cells and NK cells were decreased in COVID-19 patients with critical disease compared to those with mild COVID-19, whereas B cells and canonical monocytes were increased (Fig. 3b left column versus middle column). In patients with severe or critical COVID-19 accompanied by auto-abs to type I IFNs, the ratio of immune cells in PBMCs was similar to that in patients with mild disease (Fig. 3b left column versus right column).

Regarding the expressions of type I or type II IFN-stimulated genes (ISGs) in PBMCs, the expression of ISGs was significantly increased in the patients with critical COVID-19 without auto-abs to type I IFNs as compared to mild group (Fig. 3c-e). However, severe or critical COVID-19 patients with auto-abs did not show a profound increase of type I and/or II ISG expression (Fig. 3c-e). In particular, myeloid-derived cells from the patients with auto-abs displayed the reduced expression of type I ISG and antiviral-related genes induced by type I IFNs (Fig. S3c-e). Indeed, the expression of type I ISGs was decreased in canonical monocytes and conventional dendritic cells in patients with auto-abs (Fig. 3f). Thus, anti-type I IFN-neutralizing auto-abs attenuated type I IFN signaling in patients with severe or critical COVID-19.

### Reduced Production of SARS-CoV-2-Specific Antibodies in COVID-19 Patients with Auto-Abs to Type I IFN

**Fig. 4:**
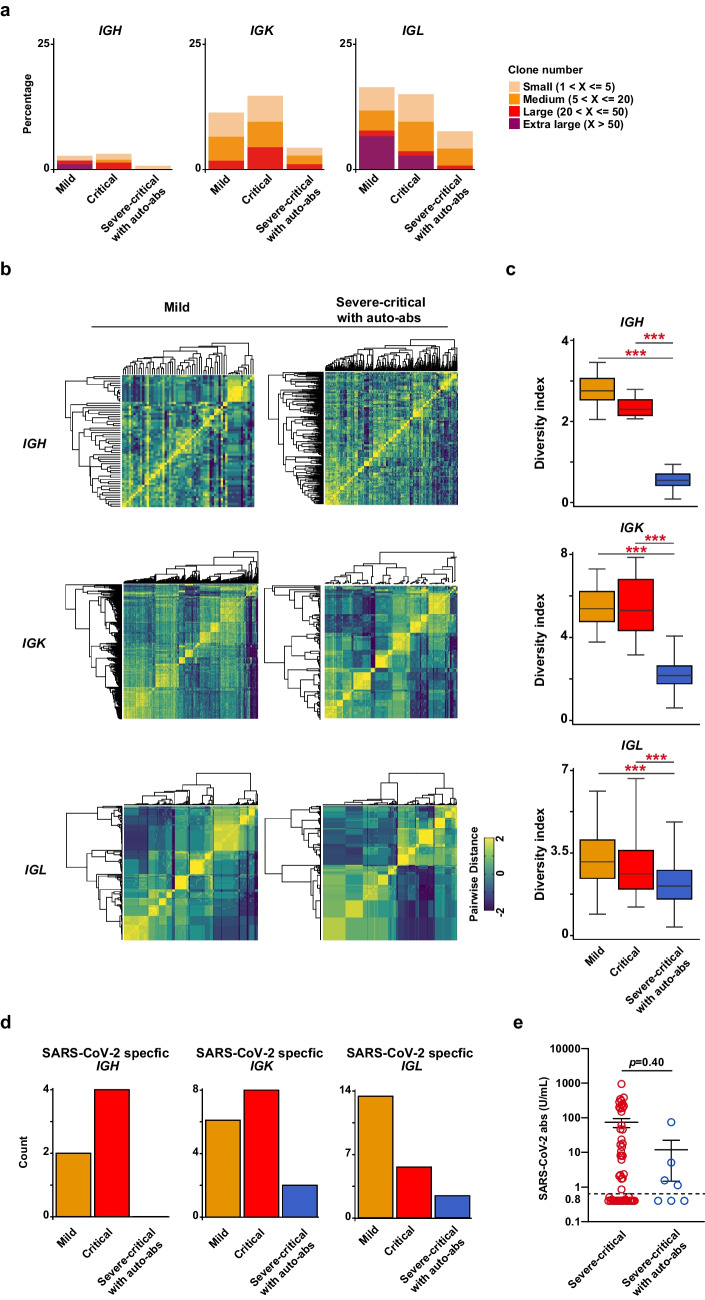
Impaired SARS-CoV-2-specific responses of B cells in COVID-19 patients with auto-abs to type I IFNs. **a**. Clone numbers of BCR repertoires (*IGH*, *IGK* and *IGL*) in CD45^+^ cells from the three groups. **b.** Pearson’s correlation coefficient for amino acid sequences converted from the scRNA-seq data of BCR repertoire (*IGH*, *IGK* and *IGL*). **c.** Diversity indexes of the clonotypes of BCR (*IGH*, *IGK* and *IGL*) in mild, critical, or severe-critical patients with auto-abs. One-way ANOVA was used for the statistical analysis. Mean ± SD. ***p <0.001. **d.** Clonotype numbers of SARS-CoV-2-specific CDR3 of *IGH*, *IGK* and *IGL* in mild, critical, or severe-critical patients with auto-abs. **e.** The concentrations of SARS-CoV-2 S protein specific antibody in the plasma. Mann-Whitney *U* test was used for the statistical analyses. Mean ± SEM

To examine whether or not auto-Abs to type IFN affect the productivity of SARS-CoV-2-specific antibody, we analyzed BCR repertoires by scRNA-Seq. The results showed a smaller clone size of B cell repertoires (*IGH*, *IGK* and *IGL*) in severe or critical COVID-19 patients with auto-abs to type I IFNs than in other patient groups (Fig. 4a). We then converted the nucleotide sequences coding CDR3 of BCR (*IGL*) into amino acid sequences and examined the diversity (Fig. 4b). The result showed that the COVID-19 patients with auto-Abs also had the lower diversity of CDR3 region in *IGH, IGK* and *IGL* (Fig. 4c). Next, to investigate whether or not the amino acid sequences of the CDR3 region in *IGH, IGK* and *IGL* were SARS-CoV-2-specific, we compared them to published data (https://opig.stats.ox.ac.uk/webapps/covabdab/). Notably, the number of SARS-CoV-2 specific clones of *IGH, IGK* and *IGL* in the COVID-19 patients with auto-abs were lower than in mild or critical patients without auto-Abs (Fig. 4d). In fact, SARS-CoV-2 S protein-specific antibodies in the plasma from patients with auto-abs tended to be lower than those in patients without auto-abs (mean: 74.3 vs 11.9 U/mL) (Fig. 4e). Thus, these results suggested that the B cell responses were impaired in patients with anti-type I IFN auto-abs.

### Recognition of Epitopes Binding to IFN α/β Receptor 1 (IFNAR1) by Anti-Type I IFN-Neutralizing Auto-Abs

**Fig. 5:**
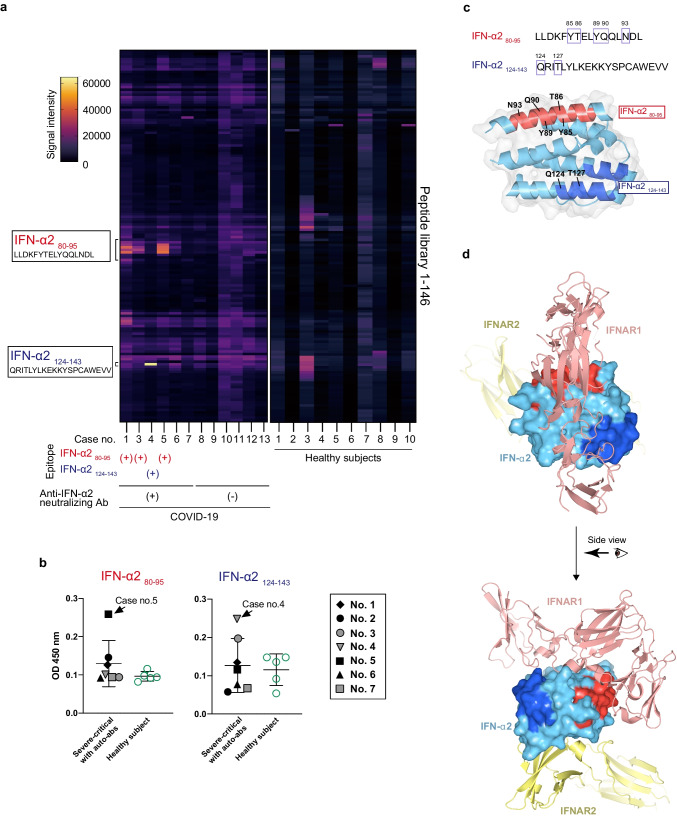
Identification of epitopes recognized by anti-IFN-α2 antibodies in COVID-19 patients. **a**. Peptide microarray against human IFN-α2 for 6 COVID-19 patients with auto-abs (Case no. 1-6), 6 COVID-19 patients without auto-abs (Case no. 8-13) and 10 healthy control subjects. The data of case no. 2 was omitted from the figure because it was not suitable for evaluation due to high background values. **b.** Detection of the auto-Abs recognizing the two specific peptides (IFN-α2 _80-95_ and IFN-α2 _124-143_) in plasma from the COVID-19 patients or healthy controls subjects by ELISA. Mean ± SD. **c.** Amino acid sequences of the two epitopes (IFN-α2 _80-95_ and IFN-α2 _124-143_) of human IFN-α2 recognized by the auto-abs (upper). The amino acids surrounded by squares indicate those that bind to the receptor. A ribbon diagram of human IFN-α2 is shown at the bottom (PDB ID: 3SE3). IFN-α2 _80-95_ and IFN-α2 _124-143_ are highlighted in red and blue respectively. **d.** Crystal structure of the human IFN-α2, IFNAR1, and IFNAR2 complex (PDB ID: 3SE3). IFN-α2, IFNAR1, and IFNAR2 are shown in light blue, pink, and yellow respectively. The red and blue colors indicate the epitopes (IFN-α2 _80-95_ and _124-143_) recognized by auto-abs found in the COVID-19 patients

Next, we investigated the epitopes of IFN-α2 recognized by the auto-abs in patients with COVID-19. To this end, we performed epitope mapping using the plasma samples from five patients with auto-abs IFN-α2 with overlapping synthetic peptides of human IFN-α2. The serum from patients with auto-abs (Case no. 1, 3, and 5) showed strong interactions with IFN-α2_80-95_ peptides, whereas that from one patient (Case no. 4) recognized IFN-α2_124-143_ peptides (Fig. 5a). To confirm these data, we performed an ELISA by coating plate with these peptides. The results showed that auto-abs in the plasma from case no. 5 and 4 recognized IFN-α2_80-95_ or IFN-α2_124-143_ peptides respectively (Fig. 5b). The sequence of IFN-α2_80-95_ is completely identical to that of IFN-α5, while the sequence of IFN-α2_124-143_ does not match that of any subtype of IFN-α (Fig. S4). These sequences included several amino acid residues with the ability to bind to IFNAR1 (Fig. 5c and 5d) [42, 43]. Furthermore, no strong interactions with any IFN-α2 peptide sequences were detected in the patients without auto-abs to IFN-α2, those with auto-abs (Case no.6 and 7) or healthy control subjects. Thus, auto-abs to IFN-α2 in these patients may have the ability to inhibit type I IFN signaling by blocking the binding to IFNAR1.

## Discussion

Type I IFNs, which inhibit viral replication in infected cells and induce anti-viral immune responses, are crucial cytokines for the initial host defense responses against virus infection [44–46]. Thus, neutralization of type I IFNs by auto-abs in the early phase of infection can increase the susceptibility to infection and result in exacerbation of the viral infection. In the case of COVID-19, many studies have demonstrated that auto-abs to type I IFN were detected in plasma or serum from patients with severe or critical COVID-19 [21, 24–28, 30, 37, 47, 48]. All of the subjects in the present study were Japanese, and the sample collection was conducted in limited areas of Japan. However, the frequencies of individuals with auto-abs to type I IFNs in severe or critical COVID-19 patients were 15.7% or 9.5% respectively, which was similar to the findings in other studies performed all over the world (10%-20% in patients with a critical or life-threatening condition) [24–30, 37, 47]. These results indicate that the prevalence of auto-abs to type I IFNs shows no obvious racial or regional discrepancy.

The prevalence of auto-abs increases with age [13, 25, 28, 47], suggesting that senescence of immunity is associated with the production of auto-abs to type I IFNs. Indeed, our study also revealed that patients with auto-abs to type I IFN were over 58 years old (Fig. 1d). Auto-abs to type I IFNs can also be observed in patients with autoimmune polyglandular syndrome, in which the autoimmune regulator gene (*AIRE*), a transcription factor that regulates the autoantigens in the thymus, is defective [49]. The expression of AIRE and self-antigen genes decreases with age in thymic B cells [50], which may result in the production of auto-abs to type I IFNs, leading to the severe form of COVID-19 in elderly patients. Furthermore, the fact that the titers of auto-abs to type I IFNs remained unchanged and retained their neutralizing activity even after treatment for COVID-19 during hospitalization indicates that the production of auto-abs to type I IFNs is due to an intrinsic defect in the immune system rather than a temporary defect caused by SARS-CoV-2 infection (Fig. 2) [21, 26, 37, 48, 51, 52].

Our scRNA-seq analysis showed that critical-severe patients with auto-abs showed no marked increase in B cells. This was accompanied by lower diversity (Fig. 3b and 4a-c). Furthermore, the numbers of SARS-CoV-2 specific *IGH, IGK* and *IGL* were reduced in the severe-critical patients with auto-abs, suggesting the impaired B cell responses against SARS-CoV-2 infection in the patients (Fig. 4d). Given that type I IFN promotes antibody production and class switching by activating B cells [46], the lower ISG expression in innate cells might lead to impaired B cell responses in patients. However, as some groups reported, type I IFN is not required for sufficient SARS-COV- 2 specific B cell responses to mRNA vaccines [53]. In addition, there is another possibility that aberrant TNFα production from Th1 cells reduces SARS-CoV-2 specific B cells by blocking T follicular helper cell differentiation [54]. Thus, further studies are needed to clarify the mechanisms underlying the impaired B cell responses in COVID-19 patients with auto-abs to type I IFNs.

Most auto-abs detected in COVID-19 patients were to IFN-α2 or IFN-ω, and auto-abs to IFN-β were very rare [21, 26, 28, 52], which was recapitulated in our study (Fig. 1). We found one COVID-19 patient with auto-abs to IFN-α2, IFN-β, and IFN-ω who died from COVID-19 pneumonia. Whether or not the existence of auto-abs to these three type I IFNs was associated with the patient’s death is unclear, but auto-abs to various type I IFNs may suppress IFN signaling more potently than in those with fewer auto-abs, resulting in a higher risk of severe disease.

Auto-abs to type I IFN in the plasma from COVID-19 patients suppressed IFN-α2-induced STAT1 activation, suggesting the possible neutralizing activity of these auto-abs to type I IFN (Fig. 1). Epitope mapping analyses revealed that the auto-abs to type I IFN in COVID-19 patients recognized the receptor binding region of IFN-α2 to IFNAR1 (Fig. 5). These results suggest that the auto-abs to type I IFN may function by blocking the binding of IFN-α2 to the receptor. The auto-abs from two patients (Case nos. 6 and 7) did not recognize any amino acid sequences of IFN-α2 but did show neutralizing activity, suggesting that they might recognize conformational (discontinuous) epitopes of IFN-α2 rather than a linear epitope.

Numerous clinical trials have shown the safety and therapeutic efficacy of IFN-α2 or IFN-β administration to COVID-19 patients [55], although treatment with IFN-α2 or IFN-β would be less effective for patients with auto-abs to type I IFNs than patients without auto-abs. Removal of auto-abs to type I IFNs by plasma exchange therapy may rescue life-threatening cases of COVID-19 [48, 51]. Therefore, the detection of auto-abs to type I IFNs in advance might be useful for providing appropriate treatment to patients with COVID-19. Furthermore, a recent study revealed that around 4% of healthy individuals over 70 years old had auto-abs to type I IFNs [13]. An analysis of the clinical data in our study revealed a few characteristic features of COVID-19 patients with auto-abs to type I IFNs (Table 1–3). Thus, it is difficult to infer from clinical data whether or not patients have auto-abs to type I IFNs. Instead, measurement of the neutralizing antibody titers is necessary to ascertain the presence of auto-abs. Recent reports demonstrated that auto-abs against type I IFN have been found in patients with influenza or middle east respiratory syndrome [21, 56–58]. Furthermore, COVID-19 patients have auto-abs not only against type I IFN but also against other cytokines and chemokines [8, 59]. Considering the possibility that other emerging viral infectious diseases may appear in the near future, large-scale screening of autoantibody carriers to type I IFNs and the other cytokines would be beneficial to reduce the risk of severe disease by administering appropriate treatment to carriers.

## Data Availability

The raw and analyzed data used for scRNA-Seq in this paper are available under the following accession number: GSE208337.

## Supplemental Information

ESM 1 (PDF 4137 kb)
