# Supplementary material for: Suppression of Type I Interferon Signaling in Myeloid Cells by Autoantibodies in Severe COVID-19 Patients
Source: J Clin Immunol. 2024 Apr 22;44(4):104. doi: 10.1007/s10875-024-01708-7 (PMC11035476; doi:10.1007/s10875-024-01708-7)
Supplement: ESM 1 — (PDF 4137 kb) [file 10875_2024_1708_MOESM1_ESM.pdf]

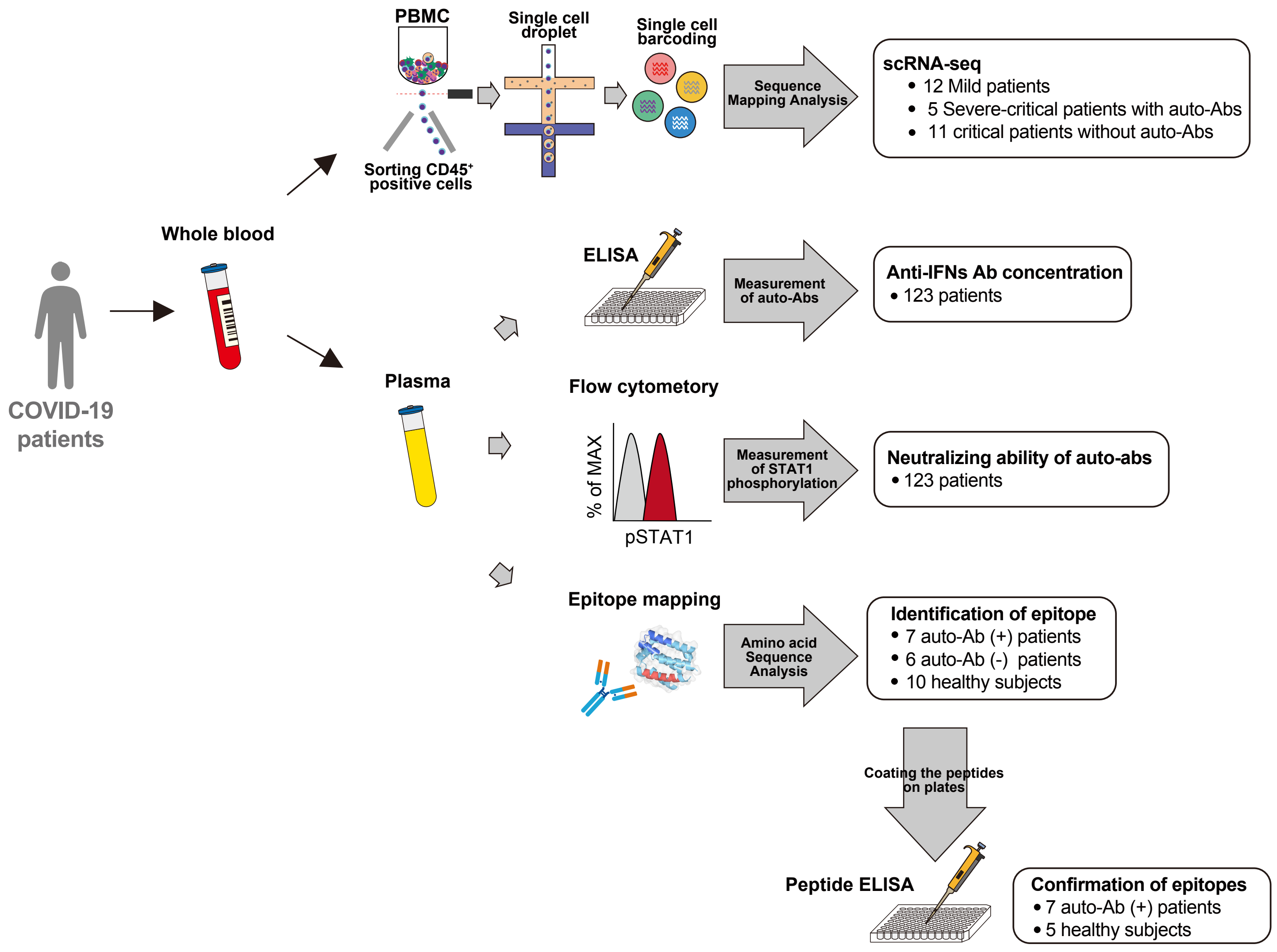

Fig. S1 Overview of the analyses in this study
Whole blood samples were collected from 123 people including healthy controls. $CD45^+$ cells were analyzed by single-cell RNA-seq. Plasma samples were used to determine the presence of neutralizing auto-abs to type I IFNs and the epitopes.

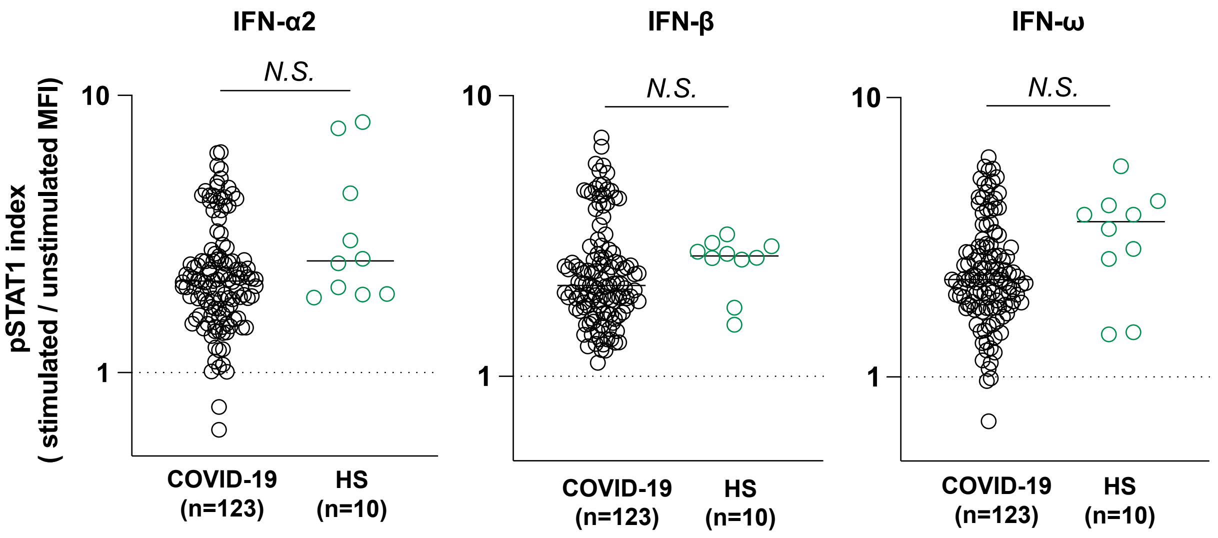

Fig. S2. Neutralizing activity to type I IFNs in plasma from COVID-19 patients
The phosphorylation of STAT1 in U937 cells stimulated with recombinant human IFN-α2, IFN-β or IFN-ω was evaluated in the presence of 10% plasma from patients (n=123) or healthy control subjects (HS, n=10). Mann-Whitney *U* test was used for the statistical analyses. The bar indicates Median.

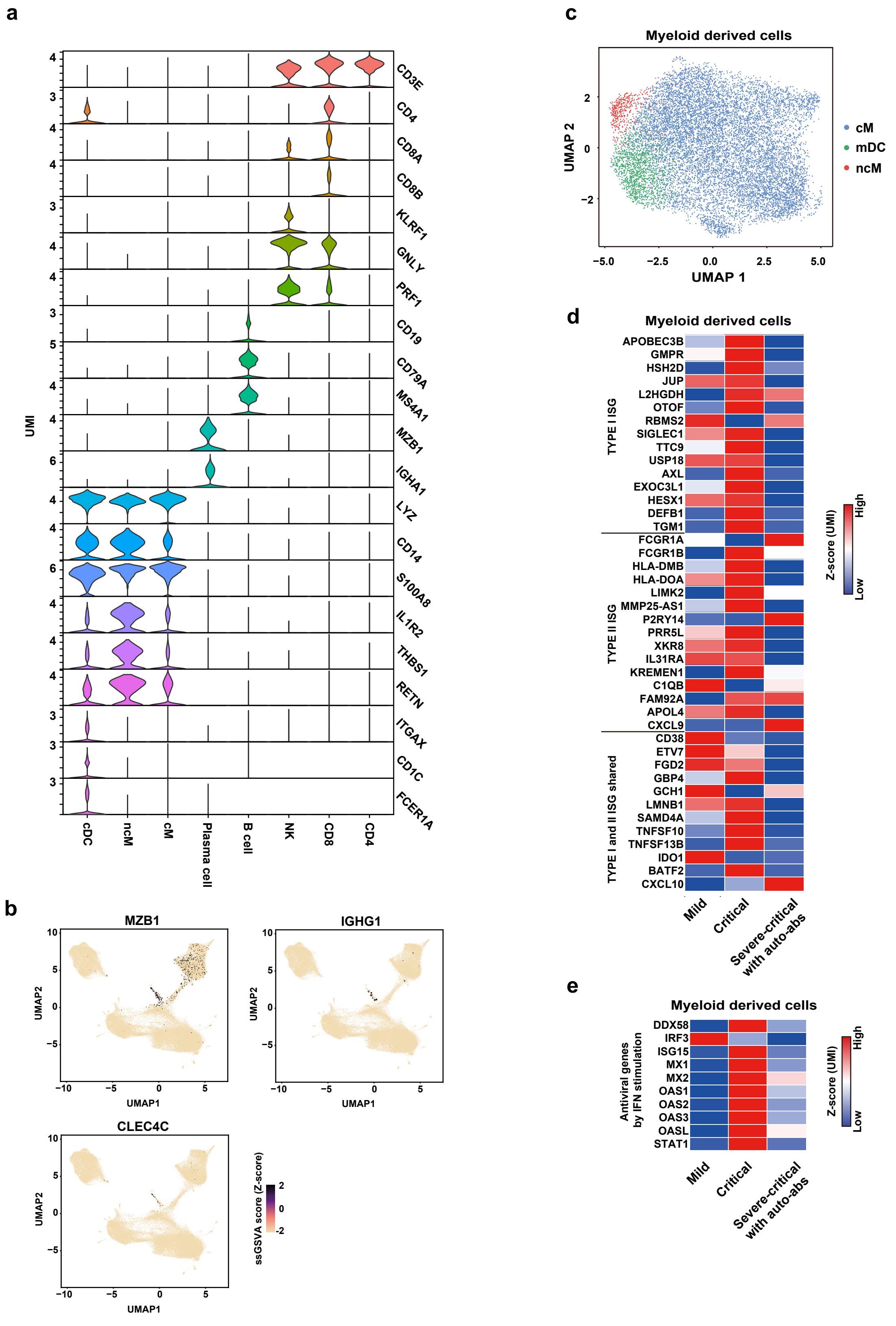

Fig. S3. Gene expression characterizing immune cells and type I IFN-related gene expression in myeloid derived cell from COVID-19 patients.

a. Violin plots depicting the expression of signature genes to identify each immune cell. **b.** The UMAP shows the expression of genes to determine plasmacytoid DCs with color intensity. **c.** The UMAP projection of CD14^{+} cells from mild, severe, and critical COVID-19 cases is depicted and colored according to the cellular populations. **d and e.** Heat map depicting the expression of ISGs (**d**) or antiviral genes induce by IFN stimulation (**e**). The color represents the Z-score for each gene.

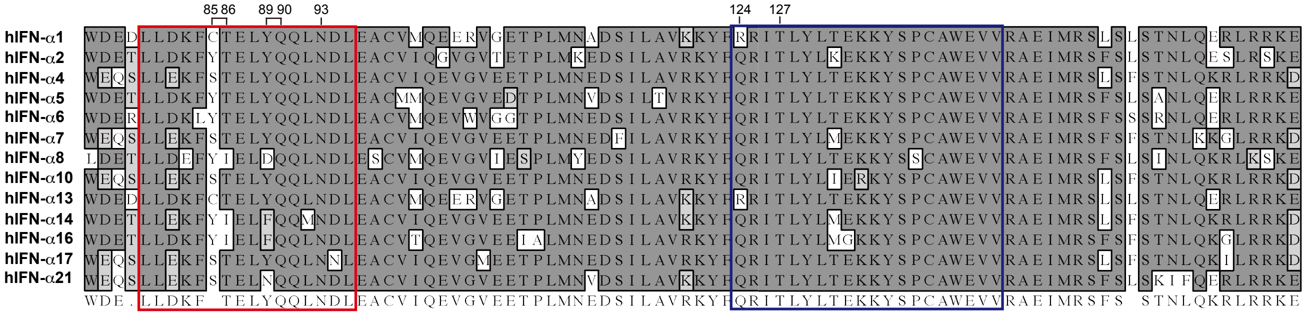

Fig. S4. A comparison of amino acid sequences between the subtypes of IFN-α
IFN-α2 80-95 is indicated by a red rectangle, while IFN-α2 124-143 is indicated by a blue rectangle. Each number on top is the binding site to IFNR1.
